# Efficacy of a Novel Nanohydrogel Formulation Containing Dopamine, Chitosan Nanoparticles, and Tridax procumbens Extract for Enhanced Wound Healing in Human Gingival Fibroblast Cells: An In Vitro Study

**DOI:** 10.7759/cureus.62819

**Published:** 2024-06-21

**Authors:** M Jeevitha, G Kaarthikeyan, Karthikeyan Ramalingam, S Rajeshkumar

**Affiliations:** 1 Periodontology, Saveetha Dental College and Hospitals, Saveetha Institute of Medical and Technical Sciences, Saveetha University, Chennai, IND; 2 Oral Pathology and Microbiology, Saveetha Dental College and Hospitals, Saveetha Institute of Medical and Technical Sciences, Saveetha University, Chennai, IND; 3 Pharmacology, Saveetha Dental College and Hospitals, Saveetha Institute of Medical and Technical Sciences, Saveetha University, Chennai, IND

**Keywords:** periodontal, tridax procumbens, chitosan nanoparticles, dopamine, nanohydrogel

## Abstract

Background

Natural compounds and biomaterials, such as nanohydrogels, have gained interest due to their biocompatibility and tissue regeneration potential. A novel nanohydrogel was prepared by employing *Tridax procumbens*, a traditional plant with anti-inflammatory properties and chitosan nanoparticles and a natural bioadhesive with potent antimicrobial and antioxidant effects and dopamine, which has been shown to regulate angiogenesis and influence cell growth. The objective of this study was to examine how human gingival fibroblast (HGF) cells respond to a nanohydrogel formulation containing dopamine, chitosan nanoparticles, and *T. procumbens* extract in terms of cell viability and cell migration.

Methods

From human gingival tissue, fibroblasts were cultured. A nanohydrogel formulation was prepared by combining dopamine, chitosan nanoparticles, and *T. procumbens* extract. Three groups were evaluated: Group 1 (nanohydrogel containing dopamine, chitosan nanoparticles, and *T. procumbens* extract (DnCTP)), Group 2 (chitosan nanoparticles and *T. procumbens* extract (nCTP)), and Group 3(*T. procumbens* extract (TP)). The MTT assay was used to measure the percentage of cell viability and a scratch assay to observe cell migration in the wounded area at different concentrations. The data were tabulated in Microsoft Excel (Microsoft Corporation, USA) and imported to IBM SPSS Statistics for Windows, version 23.0 (released 2015, IBM Corp., Armonk, NY), and the Mann-Whitney U test was conducted to statistically analyze the cell viability for different concentrations within the three groups.

Results

The nanohydrogel formulation (DnCTP) showed dose-dependent effects on cell viability with the highest cell viability at 40 µL/mL concentration, and higher concentrations of 80 µL/mL exhibited cytotoxic effects. nCTP and TP showed decreased cell viability at 80 µL/mL concentration (p < 0.05), indicating potential cytotoxicity at higher concentrations. DnCTP showed improved cell migration in the scratch assay as compared to other groups (nCTP and TP), indicating its potential for facilitating wound healing.

Conclusion

Dopamine, chitosan nanoparticles, and *T. procumbens *worked together synergistically to create a nanohydrogel formulation (DnCTP) that showed promise for improving wound healing in human gingival fibroblast cells at a dose-dependent concentration, which may therefore work as an excellent wound-healing agent in periodontal and peri-implant therapy.

## Introduction

The degenerative inflammatory changes that occur in the surrounding and supporting tissues of the tooth are referred to as periodontitis. The intricate nature of wound healing highlights its multifaceted character, where a regulated sequence of events is crucial for restoring tissue integrity and function. The main objective is to facilitate optimal wound healing while minimizing complications, adverse effects, and abnormal tissue repair. The dynamic and moist environment of the oral cavity presents difficulties in maintaining product adherence and stability [1]. Due to their biocompatibility, low toxicity, and potential to improve tissue regeneration, natural substances and biomaterials have attracted an extensive amount of interest in medical research. Nanohydrogels are nanoscale structures with a three-dimensional network of polymer chains that can absorb and hold large volumes of water or biological fluids. These nanohydrogels offer several advantages, such as flexibility, versatile behavior, and excellent biocompatibility. By stimulating a plethora of cellular and molecular scenarios that support the wound microenvironment via antibacterial, angiogenic, and anti-inflammatory activities, they exceed previously described nanosized polymers and may even shift the environment from nonhealing to healing [2].

*Tridax procumbens* (*T. procumbens*), a medicinal plant renowned for its diverse pharmacological activities, including wound-healing and anti-inflammatory properties, has been traditionally utilized in folk medicine for treating oral conditions [3]. Chitosan, a biocompatible and biodegradable polysaccharide derived from chitin, possesses antimicrobial and wound-healing properties, making it an excellent candidate for applications in tissue engineering [4]. When combined, chitosan nanoparticles and *T. procumbens* possess antimicrobial and anti-inflammatory properties and could potentially create a synergistic effect, enhancing cell proliferation and migration [5]. The therapeutic effects from the alkaloids of *T. procumbens *are amplified at the site of the wound with prolonged release of these substances.

Dopamine, a neurotransmitter is commonly associated with pleasure and reward centers in the brain. Recent research has highlighted its significance in the process of wound healing. Specifically, dopamine acts on D2 receptors and controls the regulation of angiogenesis to facilitate the regeneration of the wounded tissue [6]. In addition, dopamine influences the growth and movement of various cells involved in wound healing, such as fibroblasts and keratinocytes. Fibroblasts produce essential components like collagen and the extracellular matrix necessary for tissue repair, while keratinocytes play a crucial role in covering the wound with new skin. Furthermore, dopamine plays a significant part in marine clam adhesion, according to substantial investigations on the creatures. Moreover, dopamine enhances the bioadhesive characteristics of various coatings and hydrogels [7-8]. 

The synergistic combination of dopamine, chitosan nanoparticles, and *T. procumbens* stem extract has the potential to enhance cell viability and accelerate wound healing. This study represents the first of its kind to introduce a novel nanohydrogel formulation that combines dopamine, chitosan nanoparticles, and *T. procumbens* extract, providing a unique and innovative approach to wound healing. This formulation is based on the rationale that dopamine can improve the adhesion of the hydrogel, whereas chitosan nanoparticles may enhance cellular absorption and can deliver the agents in the hydrogel in a continuous release pattern and also speed up wound healing in human gingival fibroblast (HGF) cells.

The objective of the present study is to analyze cell viability at different concentrations of nanohydrogel containing dopamine, chitosan nanoparticles, and *T. procumbens* by MTT (3-(4, 5-dimethylthiazolyl-2)-2, 5-diphenyltetrazolium bromide) assay and to observe cell migration by a wound-healing scratch assay. The present study aims to examine how HGF cells respond to a nanohydrogel formulation containing dopamine, chitosan nanoparticles, and *T. procumbens* extract in terms of cell viability and cell migration.

## Materials and methods

Establishment of HGF primary cells

The protocol for obtaining human gingival tissue for fibroblast culture was approved by the Saveetha University Human Ethical Committee (approval no. IHEC/SDC/PERIO-2005/23/238). All patients read and signed an official consent form preceding tissue collection. Those who underwent therapeutic extraction of first or second premolars for orthodontic therapy were selected for the study. During the extraction, gingival tissues were obtained from the interdental papillae. The gingival fragments were weighed (0.18 g-0.21 g); washed with NaCl and kept in 25 cm^2^ tissue flasks filled with Dulbecco’s Modified Eagle’s Medium (DMEM) augmented with 10% fetal bovine serum (FBS), 100 U/mL of penicillin, and 100 μg/mL of streptomycin (pH 7.2; Gibco, Invitrogen, USA); and stored in a humidified CO_^2^_ incubator (5% CO_2_) at 37°C for 48 hours until cohesion of cells was noticed. To obtain principal HGF cellss, the culture media was changed two times a week for 15 days. After adding a 1X trypsin-EDTA (ethylenediamine tetraacetic acid) solution (Gibco, Invitrogen, USA), cells were extracted and used in subsequent studies. For investigations, primary HGF cells were employed between passages 4 and 5 after extraction.

*T. procumbens* stem extract

*T. procumbens* stem parts were gathered and subsequently identified. The stem pieces were meticulously dried in the shade for 10 days and then pulverized into a powder using a blender. For the extraction procedure, 50 mL of distilled water was combined with 5 g of the powdered *T. procumbens* stem and then heated for five minutes. Thereafter, the solution was filtered through Whatman filter paper to acquire the *T. procumbens* stem extract (TP).

Nanohydrogel preparation

Chitosan nanoparticles were formulated by dissolving 0.25 g of medium-molecular-weight chitosan in 0.5 mL of 1% acetic acid and 20 mL of distilled water. The solution was subjected to constant stirring using a magnetic stirrer for six hours and then stored overnight. Five mL of the *T. procumbens *stem extract that was freshly prepared was added to the chitosan nanoparticle solution to form the chitosan nanoparticles + *T. procumbens *stem extract complex (nCTP). Twelve mg of dopamine hydrochloride was dissolved in 6 mL of Tris-HCl buffer (pH 8) to create a fresh polydopamine solution (2 mg mL-1). The polydopamine solution was then added to 7.5 mL of nCTP to formulate 5% dopamine-based nanocomposite. One mL of this nanocomposite was mixed with 10 g of carboxymethylcellulose (CMC) to formulate the nanohydrogel. Three different groups were considered for further analysis: Group 1 (nanohydrogel (dopamine + chitosan nanoparticles + *T. procumbens* stem extract)- DnCTP; Group 2 (chitosan nanoparticles + *T. procumbens* stem extract- nCTP); and Group 3* (T. procumbens* stem extract- TP).

Cell viability (MTT) assay

The cell viability (MTT) assay was done for different concentrations of DnCTP (Group 1), nCTP (Group 2), and TP (Group 3). Using Dulbecco's Modified Eagle's Medium (DMEM) supplemented with 1x antibiotic solution and 10% fetal bovine serum (FBS) (Gibco, Invitrogen, USA), HGF cells were seeded at a concentration of 5 x 10^3 ^cells per well in 96-well plates. The plates were then put in a CO_^2^_ incubator that was set to 37°C and contained 5% CO_2_. The plates were rinsed with 100 μL of 1X PBS (phosphate-buffered saline). The cells were then exposed to the gel at various concentrations (5-80 μL/mL) and incubated for 24 hours at 37°C with 5% CO_2_. 0.5 mg/mL of 3-(4,5-dimethylthiazol-2-yl)-2,5-diphenyl tetrazolium bromide (MTT) dissolved with 1x PBS was added to each well. The culture plates were placed in a CO_2_ incubator and kept at 37°C for four hours. After incubation, the MTT-containing media was discarded and 200 μL of PBS was used to wash the cells. One hundred μL of dimethyl sulfoxide (DMSO) (Sigma Aldrich Chemicals Pvt Ltd, USA) were mixed with the cells, which then formed formazan crystals. Using a microplate reader, the absorbance was determined at 570 nm. The formazan dye turned to a purple-blue color, and the optical density (OD) at 570 nm was used to gauge the vitality of the cells. The fraction of viable cells was calculated using the formula: cell viability = [O.D of treated cells/ O.D of control cells] × 100

Scratch wound-healing assay

Scratch wound-healing assay was tested for the three groups: DnCTP (Group 1), nCTP (Group 2), and TP (Group 3). Six-well culture plates were seeded with HGF cells (2×10^5^ cells/well). Using an inverted microscope, a 200 μL tip was used to produce a wound in the cell monolayer, which was then cleaned with PBS, and the image was photographed. For 24 hours, the cells were treated with 20 μL/mL and 40 μL/mL concentrations of DnCTP (Group 1), nCTP (Group 2), and TP (Group 3), whereas the control group was given serum-free culture media. Using the same microscope, a picture of the scratched region was taken after a day. For every treatment group, the trials were conducted three times. The following calculation was used to quantify the percentage of wound healing based on the percentage of closure: percent closure (%) = (migrated cell surface area/total surface area) × 100.

Statistical analysis

The data were tabulated in Microsoft Excel (Microsoft Corporation, USA) and imported to IBM SPSS Statistics for Windows, Version 23.0 (released 2015, IBM Corp., Armonk, NY) for statistical analysis. Mann-Whitney U test was conducted to statistically analyze the cell viability for different concentrations within the three groups.

## Results

Cell viability (MTT) assay

**Figure 1:**
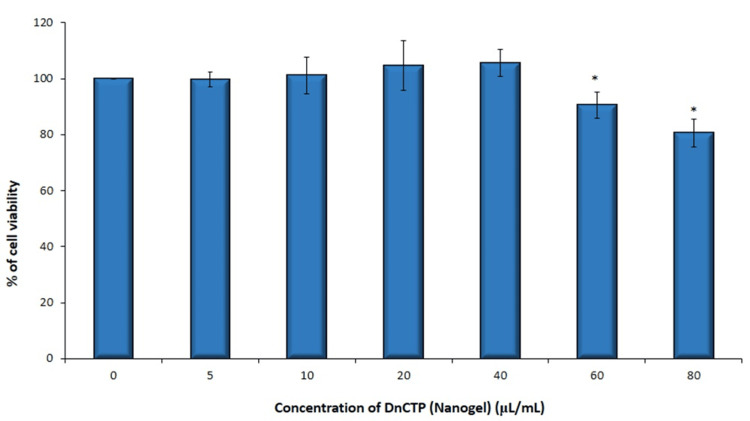
Graphical illustration showing the cytotoxic effects (cell viability %) of DnCTP nanogel (Group 1) on hGFs (human gingival fibroblasts) Cells were treated with DnCTP (nanohydrogel containing dopamine + chitosan nanoparticles + *T. procumbens* stem extract) (5-80 µL/mL) for 24 hours. Cell viability was evaluated by MTT (3-(4,5-dimethylthiazol-2-yl)-2,5-diphenyltetrazolium bromide) assay (n = 3). *Compared with the control group, p < 0.05.

The results of the MTT experiment on human gingival fibroblast cells that have been exposed to various concentrations of nanohydrogel. The mean cell proliferation was 104.6613 ± 8.801168 at 20 µL/mL, and the mean cell proliferation at 40 µL/mL was 105.6009 ± 4.916911. Cell viability percentage decreased at concentrations of 60 µL/mL and 80 µL/mL (90.52841 ± 4.685551 and 80.53209 ± 4.9381), respectively. This indicates a potential cytotoxic property of the nanohydrogel at these higher concentrations. The difference in cell viability between different concentrations of DnCTP (nanogel) was statistically significant (P = 0.020012), indicating that the cell viability was higher at 20 µL/mL and 40 µL/mL concentrations compared to other concentrations (Figure 1).

**Figure 2:**
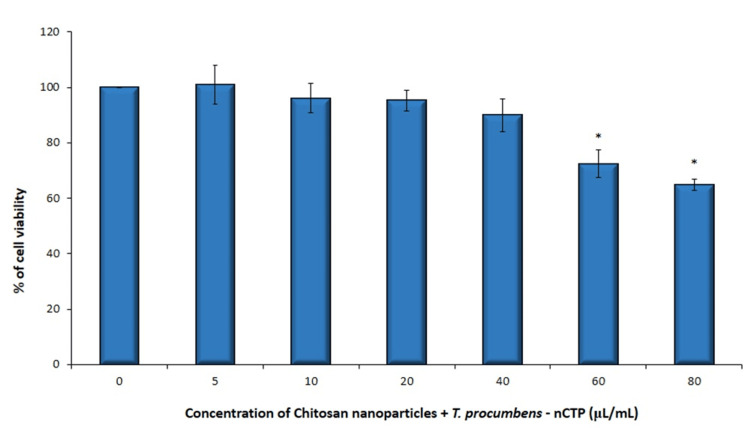
Graphical illustration showing cytotoxic effects (cell viability %) of nCTP (Group 2) on hGFs (human gingival fibroblasts) Cells were treated with nCTP (chitosan nanoparticles + *T. procumbens *stem extract) (5–80 µL/mL) for 24 hours, and cell viability was evaluated by MTT (3-(4,5-dimethylthiazol-2-yl)-2,5-diphenyltetrazolium bromide) assay (n = 3). *Compared with the control group, p < 0.05.

At lowered concentrations (5 µL/mL) of group 2 (nCTP), the cell viability was 100.92 ± 6.92. At a concentration of 10 µL/mL, there was a marginal decline in the cell viability of 96.01 ± 5.28 compared to the control. The cell viability was optimal at 20 µL/mL (95.18542 ± 5.28) and 40 µL/mL concentrations (89.87688 ± 5.28). Moreover, at concentrations of 60 µL/mL and 80 µL/mL, the cell viability was significantly decreased to 72.37 ± 4.93 and 64.84 ± 2.06, respectively, and the results were statistically significant between different concentrations (p = 0.010549) (Figure 2). These findings suggest a dose-dependent cytotoxic effect of the nCTP (*T. procumbens *extract + chitosan nanoparticles) on human gingival fibroblast cells. The higher concentrations exerted a more pronounced decline in cell viability, indicating a potential toxic effect at these concentrations.

**Figure 3:**
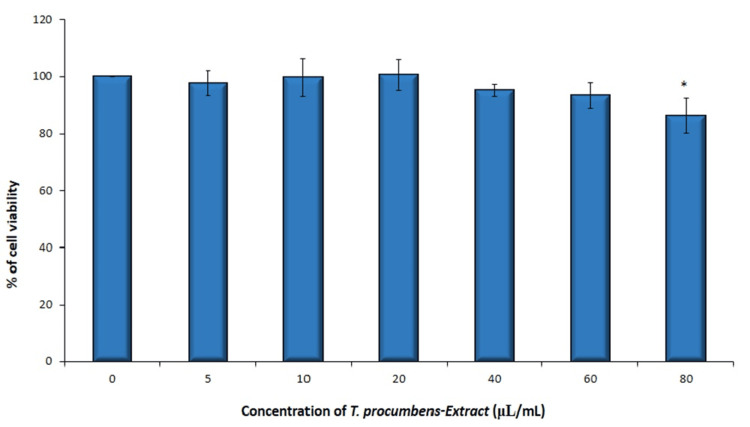
Graphical illustration showing cytotoxic effects (cell viability %) of TP (T. procumbens stem extract) (Group 3) on hGFs (human gingival fibroblasts). Cells were treated with TP extract (5–80 µL/mL) for 24 hours, and cell viability was evaluated by MTT (3-(4,5-dimethylthiazol-2-yl)-2,5-diphenyltetrazolium bromide) assay (n = 3). *Compared with the control group, p < 0.05.

In group 3 (TP), the percentage of cell viability ranged from 86.28% to 100.71% across the different concentrations. The mean cell viability was highest at 100.71% for the 20 µL/mL concentration and lowest at 86.28% for the 80 µL/mL concentration (Figure 3).

**Figure 4:**
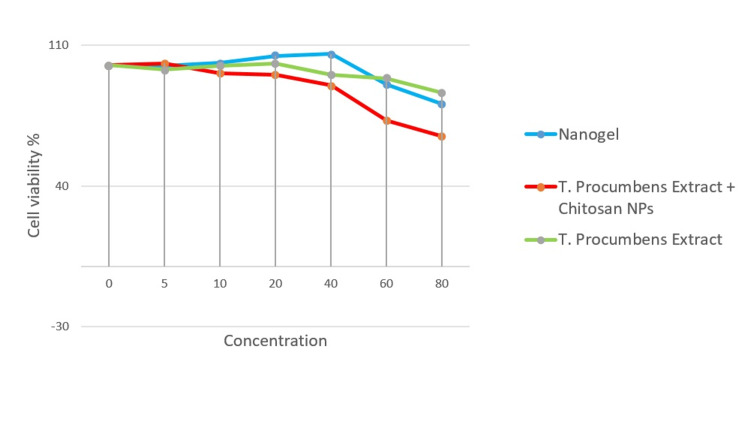
Comparison of cell viability percentage between the three groups

These findings suggest that the *T. procumbens *extract at the tested concentrations does not have a significant impact on the cell viability of human gingival fibroblast cells. This supports the notion that the extract is relatively safe and non-toxic within the given concentration range. The cell viability % decreased noticeably at 60 µL/mL and 80 µL/mL concentrations and was optimal at 20 µL/mL and 40 µL/mL concentrations for all the groups (Figure 4).

Scratch wound-healing assay

**Figure 5:**
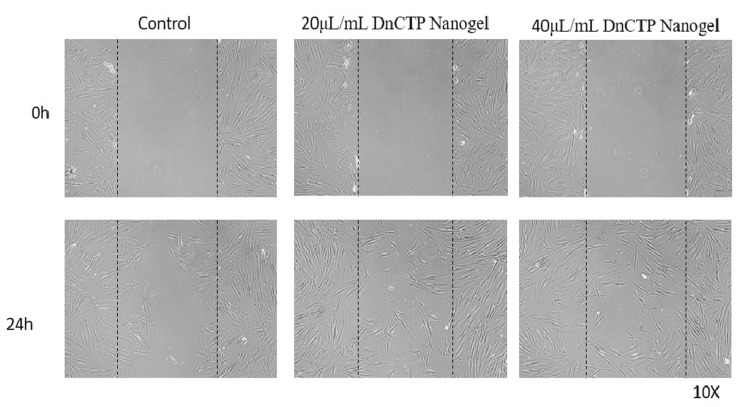
In vitro inverted microscopic image (10x) of scratch wound-healing assay of DnCTP (nanohydrogel containing dopamine, chitosan nanoparticles, and T. procumbens extract) on human gingival fibroblast cells at 20 μL/mL and 40 μL/mL concentrations

**Figure 6:**
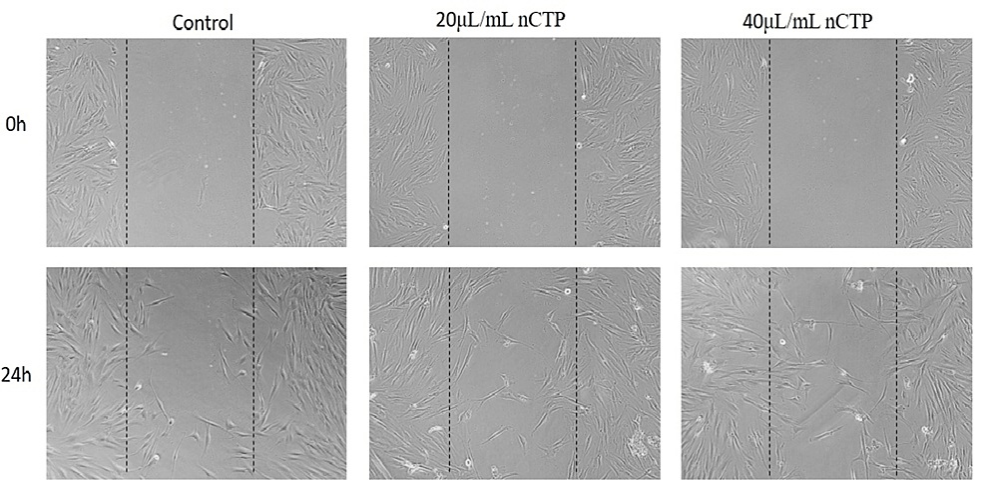
In vitro inverted microscopic image (10x) of scratch wound-healing assay of nCTP (chitosan nanoparticles and T. procumbens extract) on human gingival fibroblast cells at 20 μL/mL and 40 μL/mL concentrations

**Figure 7:**
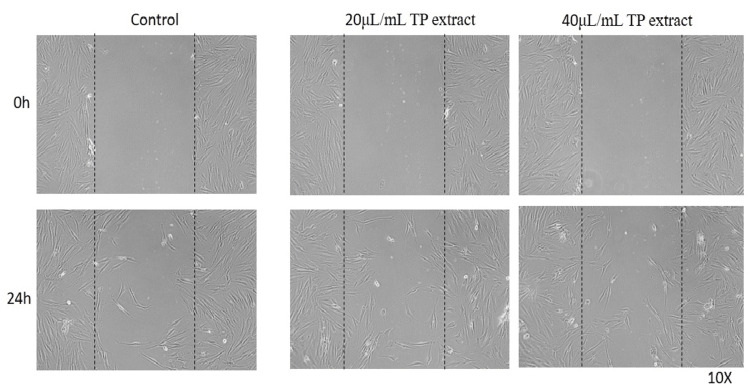
In vitro inverted microscopic image (10x) of the scratch wound-healing assay of TP (T. procumbens extract) on human gingival fibroblast cells at 20 μL/mL and 40 μL/mL concentrations

In the microscopic images, the margins of the wound were marked by dotted lines, representing the area devoid of cells in the wounded region, and the cell migration was evaluated at 20 µL/mL and 40 µL/mL for DnCTP (Group 1 (Figure 5)), nCTP (Group 2 (Figure 6)), and TP (Group 3 (Figure 7)). At the start of the experiment (0 hours), in all groups, no discernible deviations in cell migration were seen across the area of injury. However, after 24 hours, observable variations in cell migration were found.

In the 40 μL/mL concentration of nanohydrogel (group 1: DnCTP), a considerable migration of cells was observed over the dotted lines, indicating an enhanced wound-healing response compared to the group treated with 20 μL/mL nanogel. This suggests that the higher concentration of nanogel (DnCTP) had a more significant effect on promoting cell migration and wound closure. In group 1 (DnCTP), a substantial migration of cells was observed across the wounded area within 24 hours when comparing group 2 (nCTP) and group 3 (TP). The addition of dopamine to chitosan nanoparticles and *T. procumbens* extract contributed to enhanced cell migration and wound healing at 40 μL/mL concentration. This suggests that the nanohydrogel formulation, which combines dopamine, chitosan nanoparticles, and *T. procumbens* extract, exerted a significant influence on promoting cell migration and accelerating the wound-healing process.

## Discussion

The inherent resilience and targeted release potential of chitosan nanoparticles when combined with the beneficial properties of *T. procumbens* stem extracts, such as wound-healing and anti-inflammatory effects [9], made this formulation a multifunctional and synergistic intervention. Dopamine, one of the key neurotransmitters participating in various neurofunctions [10], also influences cellular proliferation [11]. It has also been shown to be susceptible to oxidation, which often hinders its effectiveness in reaching the desired target site. To address this challenge, combining dopamine with a nanoparticle, a cationic polysaccharide chitosan nanohydrogel proved advantageous [12-15]. Combining chitosan-based nanoparticles with dopamine led to increased cell proliferation and improved wound healing. The hydrophilic nature of dopamine, coupled with its high hydrogen bonding potential, further contributed to the advantages of combining it with chitosan-based nanoparticles. The evaluation of the MTT assay and scratch wound-healing assay has enhanced the understanding of the nanohydrogel's ability to promote cell survival and facilitate wound healing, which can be extrapolated in clinical applications [16].

The MTT assay results demonstrated that the nanohydrogel with dopamine had a dose-dependent effect on cell viability. At specific concentrations of 20 µL/mL and 40 µL/mL, the nanogel formulation exhibited a significant and pronounced stimulatory effect on cell proliferation. Notably, the mean cell proliferation values exceeded 100% at these particular concentrations, emphasizing the potential of the nanogel formulation at these specific doses to greatly enhance cell growth and effectively promote wound healing in HGFs.

Wang et al. similarly revealed a biphasic cytotoxic impact of dopamine, where it was cytotoxic at higher doses and cell-protective at lower concentrations. This effect could be attributed to the lower concentrations of dopamine that activated protective mechanisms in the cells, such as antioxidant defenses or anti-apoptotic pathways, counteracting the detrimental effects of oxygen-glucose deprivation/reperfusion (OGD/R) injury. Conversely, high concentrations of dopamine may have overwhelmed these protective mechanisms, leading to increased cytotoxicity and cell death. Dopamine exerts its antiapoptotic effects by activating specific signaling pathways or by modulating cellular processes involved in cell survival. Dopamine and its receptors, particularly D2 receptors, have a complex relationship in terms of both neurotoxicity (damage or harm to neurons) and neuroprotection (protection or preservation of neurons). Excessive or dysregulated dopamine signaling can lead to neurotoxicity, contributing to neuronal damage or death. On the other hand, dopamine, acting through specific receptors such as D2 receptors, may also exert neuroprotective effects by promoting cell survival mechanisms or modulating neuronal plasticity [14]. Despite the traditional role of neurotransmitters, Shome et al. postulated that dopamine has a pivotal role in angiogenesis in dermal wound tissues and subsequently in the process of wound healing. By inducing angiogenesis in the injured tissues of a mouse model, they discovered that therapy with particular D2 DA receptor antagonists dramatically improved the process of cutaneous wound healing. This could be due to the fact that dopamine causes an upsurge in the expression of HoxD3, a transcription factor important for angiogenesis, and its target, 51 integrin [6].

In the present study, the vitality of the cells in the nanohydrogel group reduced when the concentrations reached 60 µL/mL and 80 µL/mL. This finding showed that at greater doses, the nanogel formulation could be cytotoxic and appeared to have a detrimental impact on cell survival, suggesting that the cytotoxicity was dose-dependent. It was hypothesized that dopamine could cause cellular stress, compromise the integrity of the cell membrane, and trigger apoptotic pathways when their concentration crossed a particular threshold [17]. Depending on the precise formulation and characteristics of the nanogel and the kind of cells exposed to it, the severity of these cytotoxic effects could differ [18]. Thus, it was crucial to carefully optimize the concentration of the nanogel formulation to minimize cytotoxicity while still harnessing its therapeutic properties. By finding the right balance, the nanogel could effectively deliver its bioactive components without compromising cell viability or triggering undesirable cytotoxic responses.

The rapid oxidation of dopamine at physiological pH and its impact on a number of processes, including the adhesion of biomaterials, therapeutics, and conductive and adhesive material coatings, have been the subject of numerous investigations. [19,20]. In the presence of oxygen or monoamine oxidase, dopamine can self-polymerize into polydopamine [21]. A substantial amount of evidence suggested that the production of free radicals during polydopamine synthesis resulted in oxidative stress-induced cytotoxicity [22]. In particular, it was discovered that the application of antioxidants might reduce the cytotoxicity caused by dopamine-related oxidation, which in turn prompted apoptosis [23]. In addition, the creation of polydopamine prevented dopamine from attaching to thiols, amines, and hydroxyls. The difference in viability at certain concentrations was significant, which emphasized the importance of carefully selecting the concentration of the nanogel to avoid potential cytotoxic effects.

By contrast, the *T. procumbens* extract had no significant effect on the cell viability of HGFs, as indicated by the assay results. At the tested concentrations, the extract demonstrated no cytotoxic effects on cell viability. This suggested that the *T. procumbens* extract may be relatively safe and non-toxic to HGFs within the given concentration range, a finding similar to the previous literature [24,25]. The absence of significant impacts on cell viability suggested that the extract could be a suitable candidate for further research into its potential wound-healing qualities.

The scratch wound-healing assay revealed that group 1 (DnCTP) exhibited significant cell migration compared to group 2 (nCTP) and group 3 (TP), suggesting a potential synergistic effect of the nanohydrogel formulation. This may indicate that dopamine, known for its involvement in wound-healing processes, may have amplified the effectiveness of chitosan and *T. procumbens* extract, resulting in a more pronounced cell migration response [26,27]. Cell migration was crucial for recruiting different cell types to the wound site and promoting wound closure. Dopamine has been shown to enhance the movement of fibroblasts, keratinocytes, and endothelial cells involved in angiogenesis [28]. By promoting cell division, dopamine would have facilitated the timely arrival of cells to the wound site, aiding in tissue repair and the restoration of tissue integrity. Moreover, dopamine's interactions with specific receptors, such as D2 receptors, would have played a significant role in these effects [14]. The activation of D2 receptors by dopamine triggered intracellular signaling pathways that regulated gene expression, cell cycle progression, and cytoskeletal rearrangements, ultimately enhancing cell proliferation and migration.

The limitation of the present study is that it may not fully explain the mechanisms by which the nanohydrogel accelerates wound healing. Understanding the molecular pathways involved is critical for optimizing the formulation and ensuring its intended effect. It is crucial to determine the optimal concentration and dosage for safe and effective use in clinical applications as these may differ in vivo due to variances in absorption and metabolism. There might be a lack of direct comparison with existing wound-healing treatments or controls. Comparing the novel formulation with standard treatments would provide a clearer perspective on its relative efficacy and potential advantages. Addressing these limitations in future studies, particularly through comprehensive in-vivo testing and clinical trials, will be essential for validating the potential of this novel nanohydrogel formulation for wound-healing applications.

These findings provided clinical justification for the use of the nanohydrogel formulation in wound-healing applications. The incorporation of dopamine, chitosan, and *T. procumbens *extract in the nanohydrogel held promise for promoting cell migration, which was crucial for wound closure and tissue regeneration [26-29]. By facilitating cell migration, the nanohydrogel could accelerate the overall healing process, potentially leading to improved outcomes. These findings unveiled an unexplored avenue in regenerative medicine, offering promising insights into the synergistic potential of these components in enhancing wound closure and tissue regeneration.

## Conclusions

The nanohydrogel formulation demonstrated dose-dependent effects on cell proliferation, with optimal concentrations stimulating cell growth and migration. The synergistic effect of dopamine, chitosan nanoparticles, and *T. procumbens* extract showed potential in enhancing wound healing processes. These findings open up new possibilities for the development of advanced wound care therapies tailored for oral mucosal wounds.
